# Magnetocaloric Effect in Non-Interactive Electron Systems: “The Landau Problem” and Its Extension to Quantum Dots

**DOI:** 10.3390/e20080557

**Published:** 2018-07-27

**Authors:** Oscar A. Negrete, Francisco J. Peña, Juan M. Florez, Patricio Vargas

**Affiliations:** 1 Departamento de Física, Universidad Técnica Federico Santa María, Valparaíso 2340000, Chile; 2 Centro para el Desarrollo de la Nanociencia y la Nanotecnología (CEDENNA), Santiago 8320000, Chile

**Keywords:** magnetocaloric effect, magnetic cycle, thermodynamics

## Abstract

In this work, we report the magnetocaloric effect (MCE) in two systems of non-interactive particles: the first corresponds to the Landau problem case and the second the case of an electron in a quantum dot subjected to a parabolic confinement potential. In the first scenario, we realize that the effect is totally different from what happens when the degeneracy of a single electron confined in a magnetic field is not taken into account. In particular, when the degeneracy of the system is negligible, the magnetocaloric effect cools the system, while in the other case, when the degeneracy is strong, the system heats up. For the second case, we study the competition between the characteristic frequency of the potential trap and the cyclotron frequency to find the optimal region that maximizes the ΔT of the magnetocaloric effect, and due to the strong degeneracy of this problem, the results are in coherence with those obtained for the Landau problem. Finally, we consider the case of a transition from a normal MCE to an inverse one and back to normal as a function of temperature. This is due to the competition between the diamagnetic and paramagnetic response when the electron spin in the formulation is included.

## 1. Introduction

From a fundamental point of view, the magnetocaloric effect (MCE) consists of the temperature variation of a material due to the variation of a magnetic field to which it is subjected. The MCE was observed for the first time by Warburg in 1881 [1], but it was not until 1918 that Weiss and Picard [2] exposed the physical principles that govern this phenomenon, thus allowing Debye [3] in 1926 to propose the first practical applications. A year later, Giauque [4] designed magneto-thermal cycles in order to explore physical phenomena at temperatures close to the liquefaction point of helium (4.2 K). A little later in 1933, Giauque and Mac Dougall [5] reached temperatures of the order of 250 mK with paramagnetic salts exceeding for the first time in history the 1 K barrier. For more than four decades, magnetic refrigeration was confined solely to the scientific scope, thus never arriving as technology for domestic use, this being due to the low work temperature of the magnetocaloric materials. All the compounds are known to date only work at temperatures of the order of 5 K, making it impossible to use them in less extreme conditions. It was not until 1976 that Brown [6], working with a prototype of a magnetic cooler using gadolinium as the active compound, showed that it was feasible to have a refrigerating machine at room temperature. Pecharsky and Gschneider [7] discovered in 1997 a series of materials with amazing magnetocaloric responses, thus enabling the implementation of magnetothermic machines that were previously not considered feasible due to the low working temperatures. Nowadays, the research of the MCE effect has reawakened a strong interest in the scientific community again [8,9,10,11,12,13,14,15,16,17,18,19,20,21,22,23,24,25,26,27,28,29,30,31,32,33,34,35,36]. Moya et al. [21] made a recompilation of recent high temperature caloric materials and discussed other entropy-driven effects to generate thermal responses. Another interesting case is the compounds where the inverse and direct magnetocaloric effect is present [8,15], giving the possibility of a wider range of technological applications. This kind of behavior has been reproduced using theoretical models for multilayered systems under the competition of antiferromagnetic and ferromagnetic interactions [19,29,30]. Compounds with working temperatures increasingly closer to room temperature are being studied all over the world with the hope of finally finding a suitable candidate, thus replacing the current refrigeration technology by a more efficient alternative and compatible with current environmental emergencies and requirements. We highlight the work related to diamagnetic systems by Reis et al. [31,32,33,34,35,36], where they described the oscillations of the magnetocaloric effect, finding materials (especially graphene) with a strong potential application in magnetic sensors.

In physical terms, the MCE is closely linked to the behavior of the total entropy (*S*) since there is a connection between the temperature changes that a system experiences together with entropy variations. In this context, in a recent work [37], the study of the degeneracy role in the Landau problem showed a very interesting behavior for the magnetic field along an isoentropic stroke compared to the calculation in its absence. The low-temperature response of the entropy in the Landau problem is only proportional to the amplitude of the external magnetic field, so the variation of *S* is a good candidate to study the MCE for this case. On the other hand, the effects of the energy levels’ degeneracy on quantum thermodynamic quantities have been reported in many works in the past [38,39,40,41,42,43,44]. In this same framework, we highlight the work of Mehta and Ramandeep [38], who worked on a quantum Otto engine in the presence of level degeneracy, finding an enhancement of work and efficiency for a two-level particle with a degeneracy in the excited state. Furthermore, Azimi et al. [39] presented the study of a quantum Otto engine operating with a working substance of a single phase multiferroic LiCu_2_O_2_ tunable by external electromagnetic fields and that was extended by Chotorlishvili et al. [40] under the implementation of shortcuts to adiabaticity, finding a reasonable output power for the proposed machine. Therefore, the study of the role of degeneracy may be of interest to the MCE community. Besides, nowadays, it is physically possible to confine electrons in two dimensions (2D). For instance, quantum confinement can be achieved in semiconductor heterojunctions, such as GaAs and AlGaAs. At room temperature, the bandgap of GaAs is 1.43 eV, while it is 1.79 eV for AlxGa_1−*x*_ As (x=0.3). Thus, the electrons in GaAs are confined in a 1D potential well of length *L* in the *z*-direction. Therefore, electrons are trapped in 2D space, where a magnetic field along the *z*-axis can be applied [45].

On the other hand, a more realistic approach can be given if we consider an ensemble of non-interacting electrons trapped in a quantum dot, this being due to the control that can be achieved regarding the number of electrons that each quantum dot can have individually. Moreover, the advances in technology allow these systems to work below T=1 K [46,47,48,49].

In this work, we report the MCE effect for two systems: the first one corresponds to the case of the very well-known Landau problem considering the degeneracy effects in their energy levels and in the second one the case of an electron in a quantum dot subjected to a confining potential modeled as a parabolic potential in two dimensions, which is the standard approach to semiconductor quantum dots. This study is based on the fact that thermodynamics for this system can be solved by the exact calculation of the partition function, the free energy and the entropy, in such a way that the variation of the temperature with the magnetic field can be thoroughly analyzed. In particular, we found that the effect of degeneracy in the energy levels for the Landau problem modifies the magnetocaloric effect from normal to an inverse case. For an electron trapped in a quantum dot, we treat the different scenarios that were obtained due to the competition between the cyclotron frequency and the frequency related to the intensity of the parabolic trap inside the dot, finding a good inverse MCE in concordance with our results obtained for the highly degenerated Landau problem. Finally, to reinforce the idea of the role of the degeneracy on energy levels on the MCE, we consider the electron spin, thus including cases with Zeeman energy. As a consequence, there is now a competition between the diamagnetic and paramagnetic effects. This allows us to explore a broader range for the intensity of the controllable external magnetic field. Due to the splitting of energy levels and therefore a decrease in the energy levels’ degeneracy, we found MCE transitions from normal to inverse to normal form, for a particular range of temperatures and characteristic cyclotron frequency.

## 2. Model

We consider the case of an electron with an effective mass m* and charge *e* placed in a magnetic field *B*, where the Hamiltonian of this problem working in the symmetric gauge leads to the known expression (the Landau problem):

(1) $$\hat{H}=\frac{1}{2m^{*}}\left[{\left(p_{x}-\frac{eBy}{2}\right)}^{2}+{\left(p_{y}+\frac{eBx}{2}\right)}^{2}\right],$$

where we use the minimal coupling given by $\vec{p}\to \vec{p}+e\vec{A}$, being $\vec{A}$ the magnetic vector potential. The solutions for the eigenvalues of energies, which is obtained solving the Schrödinger equation, are the corresponding Landau levels of energy, the expression of which is given by:

(2) $$E_{n}=\hbar \omega_{B}\left(n+\frac{1}{2}\right).$$

**Figure 1:**
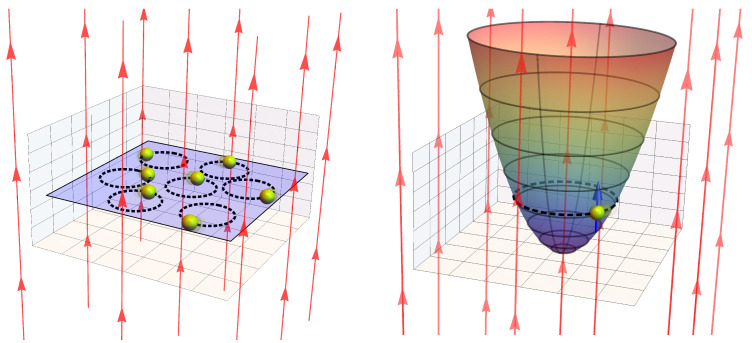
Pictorial representation of the systems. The left panel depicts the Landau problem. We recall that in our formulation, we do not consider the edge effects. Red arrows represent the external magnetic field perpendicular to the sample. The right panel depicts an electron with spin (blue arrow) trapped in a parabolic potential that represents an electron in a quantum dot.

Here, n=0,1,2,… is the quantum number, and:

(3) $$\omega_{B}=\frac{eB}{m^{*}}$$

is the standard definition for the cyclotron frequency [50,51,52,53]. With the definition of the parameter $\omega_{B}$, we can define the Landau radius that captures the effect of the intensity of the magnetic field, given by $l_{B}=\sqrt{\hbar /(m^{*}\omega_{B}})$. The energy spectrum for each level is degenerate with a degeneracy g(B) given by [53]:

(4) $$g\left(B\right)=\frac{eB}{2\pi \hbar }A,$$

with A being the area of the box perpendicular to the magnetic field *B*. Therefore, with this approach, it is straightforward to calculate the partition function for the Landau problem, and it turns out to be:

(5) $$Z_{L}=\frac{m^{*}\omega_{B}A}{4\pi \hbar }\mathrm{csch}\left(\frac{\beta \hbar \omega_{B}}{2}\right),$$

which corresponds to the standard partition function for a harmonic oscillator in the canonical ensemble, with a degeneracy of the level equal to g(B), and β corresponds to the inverse temperature $1/k_{B}T$. In Figure 1, we display a pictorial description of our studied systems.

Another interesting and highly degenerate problem corresponds to the case of a single electron trapped in a quantum dot, the non-relativistic version of which can be simply obtained if we modify the Hamiltonian of Equation (1) by adding a parabolic potential of the form:

(6) $$V\left(x,y\right)=\frac{m^{*}}{2}\omega_{d}^{2}\left(x^{2}+y^{2}\right),$$

where $\omega_{d}$ is the frequency associated with the parabolic trap and $m^{*}$ is the effective mass of the electron (Figure 1, right panel). In this case, the eigenvalues of the energy correspond to the very well-known Fock–Darwin energy levels given by:

(7) $$E_{n,m}=\hbar \Omega \left(2n+|m|+1\right)-m\frac{\hbar \omega_{B}}{2},$$

where n=0,1,2,… and m=0,±1,±2,… represent the radial and the azimuthal quantum number, respectively. The “effective frequency” Ω is defined in the form:

(8) $$\Omega =\sqrt{\omega_{d}^{2}+\frac{\omega_{B}^{2}}{4}},$$

and the effective magnetic length (or effective landau radius) is given in this case by $l_{B}=\sqrt{\hbar /(m^{*}\Omega )}$. The calculation of the exact partition function has been already discussed in previous works [50,51,53]. We highlight the works of Kumar et al. [53] and Muñoz et al. [51], where the first one calculated the partition function from a functional-integral approach and the second one by substituting the quantum numbers (n,m) for two new integer numbers $n_{+}$ and $n_{-}$. These two approaches lead to a thermodynamical system characterized by two frequencies, called $\omega_{+}$ and $\omega_{-}$, given by the expression:

(9) $$\omega_{\pm }=\Omega \pm \frac{\omega_{B}}{2},$$

and the partition function has the following form:

(10) $$Z_{d}=\frac{1}{4}\mathrm{csch}\left(\frac{\hbar \beta \omega_{+}}{2}\right)\mathrm{csch}\left(\frac{\hbar \beta \omega_{-}}{2}\right).$$

Note that the partition function has a divergence when $\omega_{d}\to 0$, but none of the thermodynamic quantities that are expressed as derivatives of $Z_{d}$ suffer from this problem. On the other hand, it happens to be that the entropy, obtained from the Equation (10), diverges for $\omega_{d}\to 0$, but we recall that this case represents a quantum harmonic oscillator of zero frequency, due to the fact that when $\omega_{d}\to 0$, we have $\omega_{-}\to 0$, and it does not represent a real physical system.

To obtain a more precise calculation, especially when we consider the case of strong magnetic fields for the electron trapped in a quantum dot, we also take into account the electron spin of value $\frac{\hbar \hat{\sigma }}{2}$ and magnetic moment $\mu_{B}$, where $\hat{\sigma }$ is the Pauli spin operator and $\mu_{B}=\frac{e\hbar }{2m^{*}}$. Here, the spin can have two possible orientations: ↑ or ↓ with respect to the applied external magnetic field *B* in the direction of the *z*-axis. Therefore, we need to add the Zeeman term in the Fock–Darwin energy levels Equation (7). Consequently, the new energy spectrum is given by:

(11) $$E_{n,m,\sigma }=\hbar \Omega \left(2n+|m|+1\right)-m\frac{\hbar \omega_{B}}{2}-\mu_{B}\sigma B.$$

The partition function for this case includes only one additional term as compared to the one given in Equation (10) and is given by:

(12) $$Z_{dS}=\frac{1}{2}\mathrm{csch}\left(\frac{\hbar \beta \omega_{+}}{2}\right)\mathrm{csch}\left(\frac{\hbar \beta \omega_{-}}{2}\right)\cosh\left(\frac{\hbar \beta \omega_{B}}{2}\right).$$

It is important to mention that this partition function, Equation (12), includes two physical effects associated with the electron trapped in the quantum dot, the diamagnetic response associated with the “csch” terms and the paramagnetism in the “cosh” term.

### Magnetocaloric Observables

To understand the expressions that we use to describe the MCE, we can think of a general non-deformable system under the action of an external magnetic field of intensity *B* at a temperature *T*, the magnetothermic properties of which can be extracted using the Gibbs free energy G. Hence, we can define the specific heat in a constant magnetic field as the second partial derivative of G with respect to temperature *T*:

(13) $$C_{B}=-T{\left(\frac{\partial^{2}G}{\partial T^{2}}\right)}_{B}.$$

Having knowledge of how the heat is transferred between the material and its environment, it is essential to understand and optimize the efficiency of the thermal machines and other systems that require the generation of temperature gradients. $C_{B}$ could give us further insights into such processes, as well as allow us to witness phase transitions between different magnetic orders as a function of different external or intrinsic parameters.

We emphasize that we work here with an entropy *S* as a function of state that depends only on two thermodynamical variables; thus, we have S≡S(T,B). This allows us to write the total differential expression for the entropy,

(14) $$dS\left(B,T\right)={\left(\frac{\partial S}{\partial B}\right)}_{T}dB+{\left(\frac{\partial S}{\partial T}\right)}_{B}dT.$$

From Equation (14), we can now derive the magnetocaloric expressions that arise from considering two thermodynamical paths, i.e., an adiabatic and an isothermal one. Correspondingly, for the adiabatic paths, we can make Equation (14) equal to zero. Using then the relation given by ${\left(\frac{\partial^{2}G}{\partial T^{2}}\right)}_{B}=-{\left(\frac{\partial S}{\partial T}\right)}_{B}$ in Equation (13) for the specific heat and the Maxwell relation ${\left(\frac{\partial M}{\partial T}\right)}_{B}=-{\left(\frac{\partial S}{\partial B}\right)}_{T}$, where the magnetization of the system has the form $M\left(T,B\right)=-{\left(\frac{\partial G}{\partial B}\right)}_{T}$, we can obtain the adiabatic change in the temperature ΔT for the system with respect to the variations of the external magnetic field; such an expression is given by:

(15) $$\Delta T=-\int_{B_{i}}^{B_{f}}\frac{T}{C_{B}}{\left(\frac{\partial M}{\partial T}\right)}_{B}dB.$$

For the isothermal path, we can obtain from Equation (14) that the change of entropy between two magnetic field is given by:

(16) $$\Delta S=\int_{B_{i}}^{B_{f}}{\left(\frac{\partial M}{\partial T}\right)}_{B}dB$$

Moreover, we can obtain the change in the entropy for a trajectory with a constant magnetic field (i.e., isomagnetic strokes) from Equation (14):

(17) $$\Delta S=\int_{T_{i}}^{T_{f}}\left(\frac{C_{B}}{T}\right)dT$$

**Figure 2:**
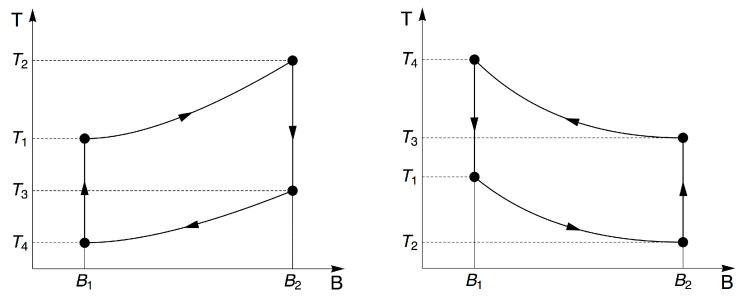
General description of the idea of the magnetocaloric effect (MCE). (Left panel) Standard MCE: We start with our system at $T=T_{1}$ and $B=B_{1}$. By performing an adiabatic stroke to $B=B_{2}$, the system heats up reaching $T=T_{2}$, the system is in contact with a thermal reservoir reaching a temperature of $T=T_{3}$. Now, we proceed with an adiabatic demagnetization stroke back to $B=B_{1}$; therefore the system cools down to $T=T_{4}$. Then the system is in contact with a sample to cool down; therefore our system reaches again a temperature of $T=T_{1}$. (Right panel): Inverse MCE: We start with our system at $T=T_{1}$ and $B=B_{1}$. By performing an adiabatic stroke to $B=B_{2}$, the system cools down to $T=T_{2}$ (this is due to the decrease of the entropy of the system’s phonons). Here, the system is in contact with a sample to cool down; therefore our system reaches $T=T_{3}$. Now, we proceed with an adiabatic demagnetization stroke back to $B=B_{1}$; therefore the system now heats up to $T=T_{4}$. The system is in contact with a thermal reservoir, therefore reaching again a temperature of $T=T_{1}$.

Now, there are two different paths for the magnetothermic cycles for the degenerate and non-degenerate cases of the Landau problem, respectively, which we describe in Figure 2. These two cases arise for the very noticeable difference in the behavior of the adiabatic trajectories discussed previously in [37]. Specifically, when we make Equation (14) equal to zero, we can construct a differential equation relating the magnetic field and the temperature for adiabatic processes in the scenario of a degeneracy, which has the form:

(18) $$\frac{dB}{dT}=-\frac{C_{1}^{2}\frac{B^{2}}{T^{3}}{\mathrm{csch}}^{2}\left(C_{1}\frac{B}{T}\right)}{\frac{1}{B}-C_{1}^{2}\frac{B}{T^{2}}{\mathrm{csch}}^{2}\left(C_{1}\frac{B}{T}\right)},$$

where $C_{1}$ is a constant given by $C_{1}=\frac{e\hbar }{2k_{B}m^{*}}$. This previous equation has an analytical solution given by:

(19) $$\begin{array}{c} C_{1}\frac{B}{T}\coth\left(C_{1}\frac{B}{T}\right)+\ln\left(C_{1}B\right)-\ln\left[\sinh\left(C_{1}\frac{B}{T}\right)\right]=C_{2}, \end{array}$$

where $C_{2}$ is an integration constant. Note that the additional term in the differential equation that provides the degeneracy, g(B), is the factor (1/B) in the dominator of Equation (18). If this term vanishes, the differential equation has a simpler form $\frac{dB}{dT}=\frac{B}{T}$ with a linear dependence between the changes of the magnetic field and the temperature, correspondingly. Because of this, we explain these two different cases separately and propose a schematic representation of the cycles for a better understanding. It is important to recall that these two proposed cycles that we discuss next are two of the many cycles that can be envisioned for magnetocaloric heating or cooling to exploit direct or inverse MCE, and the reason for showing them is to reinforce the idea of the different behaviors that take the system under the external magnetic field, in the isoentropic trajectories, due to the degeneracy effect.

*Non-degenerate case:* In this case (Figure 2, left panel), the system, having a temperature of $\left(T_{1}\right)$, is magnetized adiabatically; therefore, the final temperature $\left(T_{2}\right)$ is greater than the initial one $\left(T_{2}>T_{1}\right)$. At this point, the system is put in contact with a cold reservoir so that it reaches a lower temperature $\left(T_{3}<T_{2}\right)$. From there, an adiabatic demagnetization occurs, cooling the system again, reaching a final temperature of $\left(T_{4}\right)$, which is less than the initial temperature of $\left(T_{4}<T_{1}\right)$. Here, we use a system to cool a sample (gas or solid material), then the system reaches a final temperature of $(T_{5}=T_{1}$ (theoretically)), and therefore, our magnetic system is heated up again beginning with the initial temperature $\left(T_{1}\right)$, and a new cycle starts.

*Degenerate case:* In this case (Figure 2, right panel), our magnetic system is at temperature $T_{1}$. When we subject the system to an adiabatic magnetization, the system reaches a temperature of $T_{2}<T_{1}$. At this moment, we use our system to cool a sample (gas or solid material); therefore, this adds heat to our magnetic system, reaching a temperature of $T_{3}$$\left(T_{3}>T_{2}>T_{1}\right)$. Now, we start from here, performing an adiabatic demagnetization process, and our system therefore reaches a final temperature of $T_{4}$, higher than the temperature $T_{3}$. At that moment, the system should be put in contact with a reservoir at temperature $T_{1}$ or less in order to reach that temperature quickly, so that the cycle can be started again. Thus, we are able to cool a material.

For the case of an electron trapped in a quantum dot, we treat two instances: the case with and without an intrinsic spin. We report that, if we do not consider the Zeeman effect, the system responds like in the degenerate case of the Landau problem. When the Zeeman term is taken into account, the system’s experiment reflects both direct and inverse MCE.

It is important to recall that in our thermodynamic analysis, all the thermal quantities are derived from the partition function Z. In the generic form:

(20) $$S\left(T,B\right)=k_{B}T{\left(\frac{\partial \ln Z}{\partial T}\right)}_{B},$$

(21) $$C_{B}={\left(\frac{\partial U}{\partial T}\right)}_{B},$$

where $U=k_{B}T^{2}{\left(\frac{\partial \ln Z}{\partial T}\right)}_{B}$ and, finally:

(22) $$M=k_{B}T\left(\frac{\partial \ln Z}{\partial B}\right).$$

Before presenting the results, it is important make a clarification. Our adiabatic paths are classical, that is where the process is identified in terms of the conservation of the entropy and the isolation of the system from heat exchange with the thermal bath [37]. We recall that we work in a semi-classical scenario where the quantum part is related to the nature of the working substance and the classical part is due to the condition imposed over the adiabatic strokes along the MCE that we propose. We emphasize that the MCE is (until now) a classical effect.

## 3. Results and Discussion

### 3.1. Landau Problem: Influence of Energy Degeneracy on the MCE

From Equation (5), we straightforwardly calculated the thermodynamic quantities for the non-degenerate case. First, we analyzed the MCE starting with the corresponding thermodynamic entropy, which is given by:

(23) $$S\left(T,B\right)=\frac{\hbar \omega_{B}}{2T}\coth\left(\frac{\beta \hbar \omega_{B}}{2}\right)+k_{B}\ln\left[\mathrm{csch}\left(\frac{\beta \hbar \omega_{B}}{2}\right)\right]$$

**Figure 3:**
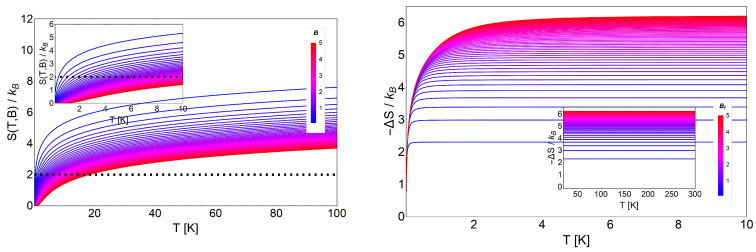
(Left panel) Entropy (in $k_{B}$ units) for the non-degenerate case of the Landau problem, as a function of temperature (in Kelvin) and for different values of external magnetic field intensity (measured in Tesla) from 0.1 T to 5 T. The straight horizontal line represents the adiabatic line $S\left(T,B\right)/k_{B}=2$, cutting different magnetic fields’ entropies at different temperatures. The inset shows the entropy in the temperature range from T=0 K to T=10 K. (Right panel) Entropy change, −ΔS, according to Equation (16), as a function of the temperature for the non-degenerate case, where $\Delta B=B_{f}-B_{i}$. We display this figure with an initial value of the external magnetic field of $B_{i}=0.01\;T$ to $B_{f}$ (from 0.1 T to 5 T) and for temperatures from T=0.01 K to T=10 K. The inset depicts the values for ΔS from T=40 K to T=300 K.

**Figure 4:**
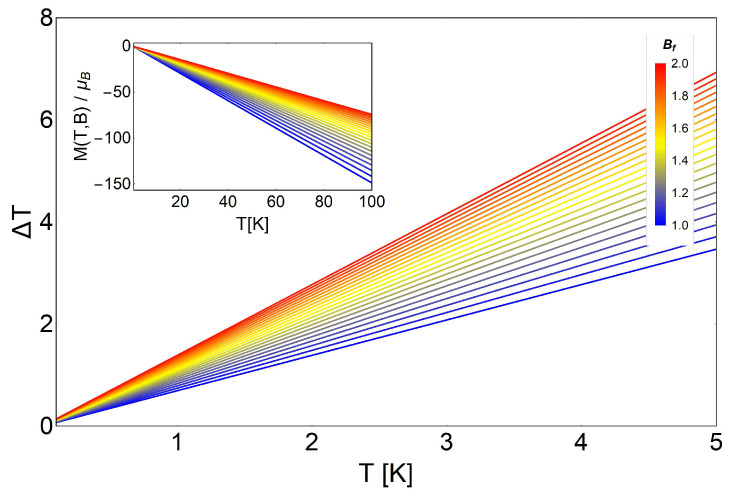
MCE for the non-degenerate case of the Landau problem as a function of temperature. We display ΔT with an initial value of the external magnetic field $B_{i}=$ 0.5 T to $B_{f}$ from 1 T (blue) to 2 T (red). The inset shows the values for M(T,B) for external magnetic fields ranging from B= 1 T to B= 2 T.

The entropy as a function of the temperature shows two behaviors. First, it grows as a function of the temperature, and second, it decreases at a fixed temperature when the intensity of the external magnetic field increases, as we show in the left panel of Figure 3. If we consider an isoentropic trajectory, represented for example by a straight horizontal line in Figure 3, as the magnetic field increases, the different curves of the entropy cut the straight horizontal line at successively higher temperatures. This explains the linearity that we obtained from the solution of the trivial differential equation of first order given in Equation (18) when we do not have the extra term 1/B in the denominator of the same equation. In the right panel of Figure 3, we observe that for all temperature ranges, the values for −ΔS are positive, so we expect a normal MCE, as we see in Figure 4 with corresponding positive values for ΔT. In real low dimensional systems, just small changes of temperature are often achieved. If we use the above argument, that would mean that the usual work temperatures are also small, as indeed they are. To obtain a realistic value for ΔT, we need to work at low temperatures. This is a consequence of the linear relationship between *B* and *T* in the adiabatic paths for this ideal system. The physical reason for this is that we are dealing with an ideal and perfect diamagnetic system. In particular, for this section, we use the regular mass for the electron corresponding to $m_{e}\equiv m^{*}$ = 9.1094 ×$10^{-31}$ kg.

The magnetization for the non-degenerate case is simply given by the expression:

(24) $$M\left(T,B\right)=-\mu_{B}\coth\left(\frac{\hbar \omega_{B}}{2k_{B}T}\right),$$

where $\mu_{B}=e\hbar /2m^{*}$ is the Bohr magneton. We plot this magnetization behavior in the inset figure of Figure 4 in the range of low temperatures and low magnetic fields. As can be appreciated, the absolute value of the magnetization decreased as the external magnetic field increased. The explanation of this conduct is the diamagnetic response. We need to remember that we have spinless electrons, so the magnetization is associated with a circular constant current *I* (charge times velocity) of section *A* in the form of M∼IA, where *A* can be written as:

(25) $$A=\pi l_{B}^{2},$$

where $l_{B}$ is the Landau radius. Therefore, the higher the magnetic field, the smaller the effective area *A* is, in order to localize the electron in a smaller region in space and vice versa. Therefore, the magnetization decreases by increasing the external magnetic field, as we see in the inset of Figure 4.

**Figure 5:**
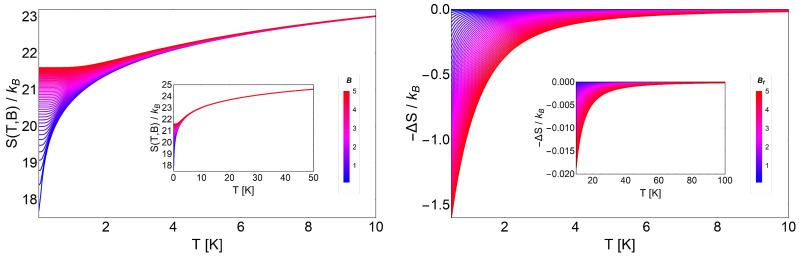
(Left panel) Behavior of the entropy $S_{L}\left(T,B\right)$ for the Landau problem with degeneracy. We show $S_{L}\left(T,B\right)$ in the range of *B* between 0.1 T to 5 T and for a temperature up to 10 K (the area used was A≡1 mm^2^). It is clearly observed that the entropy grows with the magnetic field and approximately collapses to the same value at high temperatures, as we see in the inset graphic. (Right panel) $\Delta S_{L}$ for the degenerate scenario of the Landau problem. We show $-\Delta S_{L}\left(T,B_{i},B_{f}\right)$ in a range of the external magnetic fields, for $B_{i}=$ 0.01 T and $B_{f}=$ 0.1 T to $B_{f}=$ 5 T and up to 10 K in temperature. The inset figure shows the variation of entropy for a range of temperatures between 10 K and 100 K, where we can clearly observe that the variation of −ΔS decreases approximately by a factor of 100 as compared to the low temperature behavior, as expected.

The effect of the degeneracy in the energy levels of the Landau problem modifies all the results previously presented. The entropy for this case is given by:

(26) $$S_{L}\left(T,B\right)=\frac{\hbar \omega_{B}}{2T}\coth\left(\frac{\beta \hbar \omega_{B}}{2}\right)+k_{B}\ln\left[\frac{g(B)}{2}\mathrm{csch}\left(\frac{\beta \hbar \omega_{B}}{2}\right)\right],$$

where this expression only differs from Equation (23) in the logarithmic term by the additional factor g(B)/2. This entropy is shown in the left panel of Figure 5, where we clearly see an important feature at low temperatures, i.e., the entropy of the Landau problem with degeneracy satisfies the following relation:

(27) $$S_{L}{\left(T,B\right)}_{T\to 0}\equiv S\left(B\right)∼k_{B}\ln\left(g(B)\right).$$

This entropy depends only on the external magnetic field and at T=0 simply represents the residual entropy of the ground state. It grows if the intensity of the external field grows, contrary to the non-degenerate case. This can be explained by the fact that for low temperatures, the strong linear degeneracy dependence on the field implies more available states, so consequently, the entropy must increase as the field increases. On the other hand, in the high temperature range (see the inset in the left panel of Figure 5), the entropy for different magnetic fields approximately collapses to the same value, but there still exists a finite ΔS>0, allowing the analysis of the MCE at higher temperatures.

**Figure 6:**
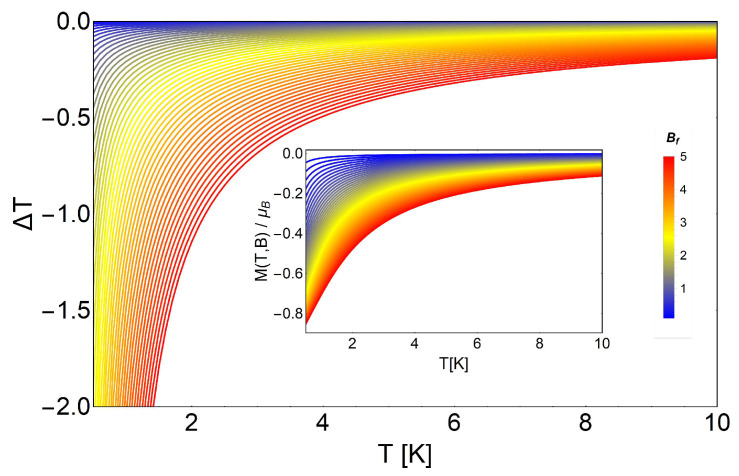
MCE for the Landau problem with degeneracy as a function of temperature. The main figure shows ΔT in a range of the external magnetic fields, from $B_{f}=$ 0.1 T to $B_{f}=$ 20 T at fixed $B_{i}=$ 0.01 T, and up to 10 K in the temperature scale. The inset shows the values for M(T,B) for external magnetic fields ranging from B= 0.01 T to B= 5 T.

As we see in the right panel of Figure 5, we obtain negative values for −ΔS, opposite to the non-degenerate case (see Figure 3). Therefore, when we analyze the MCE for this system, we obtain negative values for ΔT, as presented in Figure 6. Here, we discuss two possible scenarios: low temperature and high temperature behavior for the same range of external magnetic fields. In the case of low temperature, we obtained larger values for ΔT, but it is important to point out that we had only a region of physical validity to avoid negative temperatures. Approximately from a temperature of T∼2 K and above, we can work with all the proposed range of magnetic fields (from B= 0.01 T to B= 5 T), as we see in Figure 6, because all the values of ΔT were acceptable (i.e., $T_{f}=T_{i}+\Delta T>0$ K). If we decrease the value of the initial temperature *T* beyond this value, we must proceed carefully. At lower temperature, the physically acceptable solutions cannot take higher values for the external magnetic field in order to avoid final negative temperatures, as we clearly appreciate in Figure 6.

### 3.2. MCE for Electrons Trapped in a Quantum Dot

In this section, we now develop the case of an electron trapped by a quantum dot. From Equation (10), it is straightforward to derive the entropy, $S_{d}\left(T,B\right)$, for this problem:

(28) $$\begin{array}{cc} \frac{S_{d}\left(T,B\right)}{k_{B}} & =\frac{\hbar \omega_{+}}{2k_{B}T}\coth\left(\frac{\hbar \omega_{+}}{2k_{B}T}\right)-\ln\left(2\sinh\left(\frac{\hbar \omega_{+}}{2k_{B}T}\right)\right)\\ & +\frac{\hbar \omega_{-}}{2k_{B}T}\coth\left(\frac{\hbar \omega_{-}}{2k_{B}T}\right)-\ln\left(2\sinh\left(\frac{\hbar \omega_{-}}{2k_{B}T}\right)\right). \end{array}$$

**Figure 7:**
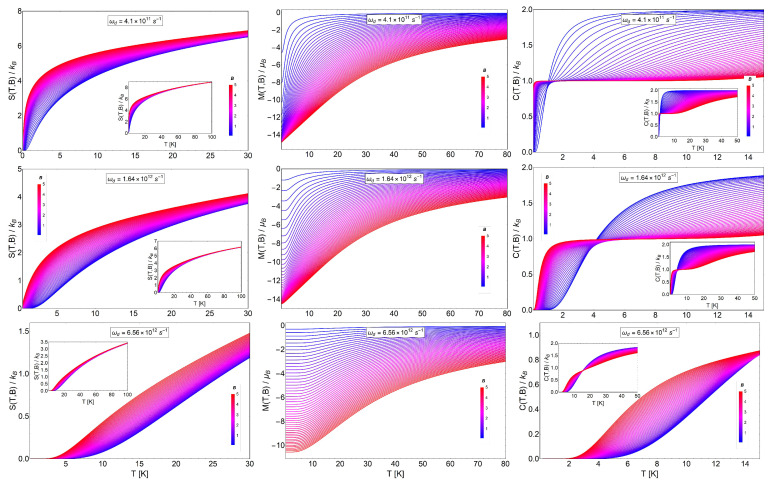
Spinless electrons in a quantum dot. Entropy $S_{d}\left(T,B\right)$ (left panels), M(T,B) (middle panels) and $C_{B}\left(T,B\right)$ (right panels) as a function of temperature (T) from 0 K to 30 K for different values of magnetic fields in the range of 0.1 T to 5 T. In the middle panels of the graphs, we selected the representative value for the characteristic frequency of the harmonic trap in $\omega_{d}=1.6407\times 10^{12}$ s^−1^, which in terms of energy represents an approximate value of 1.07 meV. For the top panels, we use the value $\frac{\omega_{d}}{4}$ and in the bottom panels the case of $4\omega_{d}$ for the characteristic frequency of the dot structure ($\omega_{B}=\frac{eB}{0.067m_{e}}\equiv 2.63\times 10^{12}\;B$ s^−1^, where *B* is in Tesla units for comparison).

Figure 7 shows the entropy, magnetization (from Equation (22)) and specific heat (from Equation (21)) as a function of temperature. We clearly appreciate that the entropy function grows with the magnetic field as in the case of the Landau problem with the degeneracy effects. Thence, by calculating $-\Delta S=S\left(T,B_{i}\right)-S\left(T,B_{f}\right)$ with $B_{f}>B_{i}$, we obtain negative values. This result was expected due to the strong degeneracy of the Fock–Darwin levels reflected in the dependence of the spectrum of Equation (7) in the azimuthal quantum number *m*. The partition function captures this effect when we write the quantum numbers (n,m) in the form of two new numbers $\left(n_{+},\;n_{-}\right)$ in order to form two frequency oscillators given by $\omega_{+}$ and $\omega_{-}$. For the next two sections in the manuscript, we consider an effective mass $m^{*}∼0.067$ m_*e*_. This effective mass is associated with a cylindrical quantum dot of GaAs with a typical radius of 20 to 100 nm [54,55]. For the characteristic frequency of the trap $\omega_{d}$, we use the value of $1.6407\times 10^{12}\;s^{-1}$, which in terms of energy represents approximately $\hbar \omega_{d}∼1.07$ meV. The selection of this particular value is to compare the intensity of the trap with the typical energy of intra-band optical transition of the quantum dots. The order of this transition is approximately around ∼1 meV for GaAs quantum dots [54].

**Figure 8:**
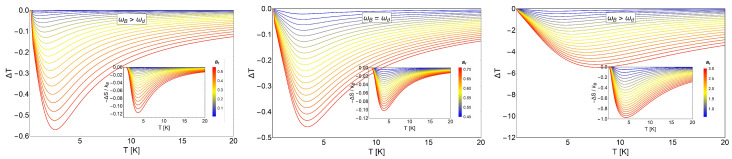
MCE effect for spinless electrons in a quantum dot. ΔT for: (i) $\omega_{B}<\omega_{d}$ (left panel), (ii) $\omega_{B}∼\omega_{d}$ (middle panel) and (iii) $\omega_{B}>\omega_{d}$ (right panel). The insets of all graphics in this figure: −ΔS in units of $k_{B}$ as a function of temperature for: (i) $\omega_{B}<\omega_{d}$ (left panel), (ii) $\omega_{B}∼\omega_{d}$ (middle panel) and (iii) $\omega_{B}>\omega_{d}$ (right panel). For all graphics presented in this figure, we have selected the value of $\hbar \omega_{d}∼1.07$ meV (i.e., $\omega_{d}=1.6407\times 10^{12}$ s^−1^).

For the analysis of the ΔS, it is convenient to analyze the results in three cases due to the competition between $\omega_{d}$ and $\omega_{B}$. In order to do this, we plot the variation of the entropy as a function of temperature for the case of a characteristic dot frequency of $\omega_{d}=1.6047\times 10^{12}\;s^{-1}$, the value of which is associated with a cyclotron frequency for B= 0.625 T (i.e., $\omega_{B}=2.63\times 10^{12}\;B\;s^{-1}$, with *B* in units of Tesla) of the external magnetic field intensity. Thereby, to analyze the different regions of −ΔS, we plot this quantity in the three insets of Figure 8 using an initial value of the external magnetic field of $B_{i}=$ 0.01 T, $B_{i}=$ 0.45 T and $B_{i}=$ 0.61 T in the left, center and right panel, respectively. This is in order to satisfy the three regimes of frequencies. The left panel is associated with a range of final external fields corresponding to $B_{f}$ between 0.02 T and 0.49 T (i.e., $\omega_{d}>\omega_{B}$), the middle panel a range of final external fields corresponding to $B_{f}$ between 0.49 T and 0.71 T $\left(\omega_{d}∼\omega_{B}\right)$ and the right panel for $B_{f}$ from 0.72 T to 3 T (i.e., $\omega_{d}<\omega_{B}$). We observe that the magnitude of −ΔS increases (in absolute value) for higher magnetic fields, as we see from the diagram for S(T,B) of Figure 7, and is more pronounced in the range of temperature from T∼2 K to T∼4 K.

For the MCE observable ΔT, in coherence with the values of −ΔS, we obtained a major increase at higher values of the external magnetic field. At a field value of B= 3 T, we approximately obtained ΔT of 5.5 K (in absolute value). This can be appreciated in the right panel of Figure 8. We recall that we obtained an inverse MCE due to the strong degeneracy of this problem, and this is in concordance with the result previously discussed for the case of the Landau problem with degeneracy.

### 3.3. MCE for Electrons with Spin Trapped in a Quantum Dot

In this case, the entropy has the following form as displayed in Equation (12):

(29) $$\begin{array}{c} \frac{S_{dS}\left(T,B\right)}{k_{B}}=\frac{S_{d}\left(T,B\right)}{k_{B}}+\ln\left(2\cosh\left(\frac{\hbar \omega_{B}}{2k_{B}T}\right)\right)-\frac{\hbar \omega_{B}}{2k_{B}T}\tanh\left(\frac{\hbar \omega_{B}}{2k_{B}T}\right), \end{array}$$

where $\frac{S_{d}\left(T,B\right)}{k_{B}}$ is given by Equation (28). We can see two new terms in the last expression for the entropy when comparing to the previous case for Equation (28). These terms represent the paramagnetic contribution to the entropy arising from the spin coupled to the magnetic field. We will see below that they play a fundamental role in the results for the MCE. As the entropy of a paramagnet vanishes as a function of the magnetic field at any finite temperature, the behavior of −ΔS will always be positive for the paramagnetic contribution.

**Figure 9:**
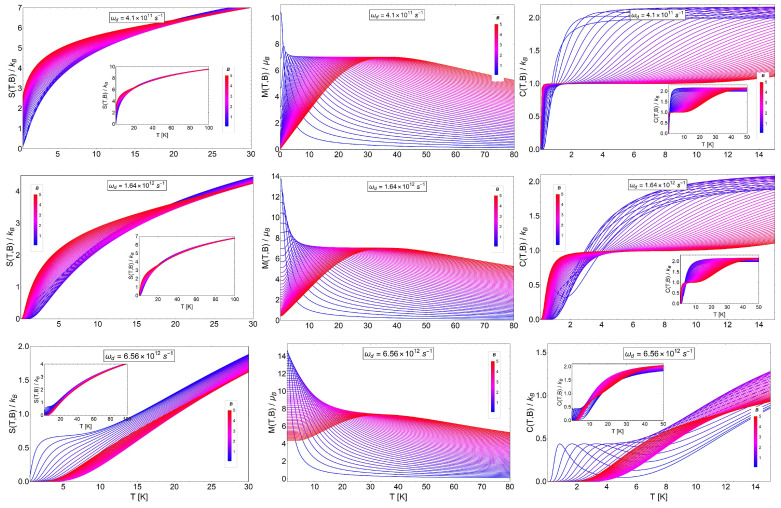
Electrons with spin trapped in a quantum dot. Entropy $S_{dS}\left(T,B\right)$ (left panel), M(T,B) (middle panel) and $C_{B}\left(T,B\right)$ (right panel) as a function of temperature (T) from different regions of temperature between 0 K to 80 K for different values of the magnetic field in the range of 0.1 T to 5 T. In the middle panels of the graphs, we selected the representative value for the characteristic frequency of the harmonic trap in $\omega_{d}=1.6407\times 10^{12}$ s^−1^, which in terms of energy represents an approximate value of 1.07 meV. For the top panels, we use the value $\frac{\omega_{d}}{4}$, and in the bottom panels, the case of $4\omega_{d}$ for the characteristic frequency of the dot structure is used. The insets show the thermodynamic quantities (in the left and right panel) in an extended temperature range between T=0 K and T=100 K.

In Figure 9, we show the entropy (Equation (29)), magnetization (from Equation (22)) and specific heat (from Equation (21)) as a function of temperature. We see in the figure different behaviors of these thermodynamic quantities as a function of temperature for different magnetic field ranges. We considered three different characteristic dot frequencies as we did before in the case of the spinless electron in the quantum dot. In the middle panels, we consider the characteristic dot frequency of $\omega_{d}=1.6407\times 10^{12}\;s^{-1}$, the value of which can be assimilated as a cyclotron frequency for B= 0.625 T (i.e., $\omega_{B}=2.63\times 10^{12}\;B\;\;s^{-1}$). For the top panel of Figure 9, we use the value $\frac{\omega_{d}}{4}$, and for the figures in the bottom panels, we use the value of $4\omega_{d}$ for the characteristic frequencies of the dot structure. The insets (for the left and right panel) show the same quantities in an extended temperature range, from T=0 K to T=100 K.

**Figure 10:**
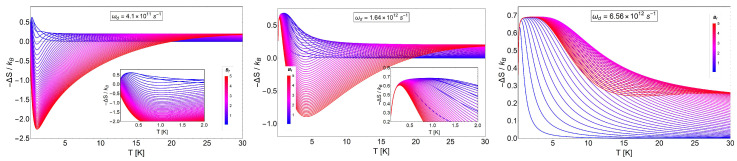
$-\frac{\Delta S_{dS}}{k_{B}}$ as a function of temperature for different values of characteristic dot frequencies. The middle panel corresponds to a value of $\omega_{d}=1.6407\times 10^{12}$ s^−1^. For the left panel, we selected the value of $\frac{\omega_{d}}{4}$ and for the right panel the value of $4\omega_{d}$. The range of the external magnetic field values, $B_{f}$, is between 0.02 T and 5 T, and $B_{i}=$ 0.01 T. The temperature range is from T=0.01 K to T=30 K.

In Figure 10, we show the quantity $-\Delta S_{dS}$ for three characteristic dot frequencies $\omega_{d}$. We clearly observe a transition for $-\Delta S_{dS}$, from positive to negative and back to positive values, indicating that we have different MCE effects.

**Figure 11:**
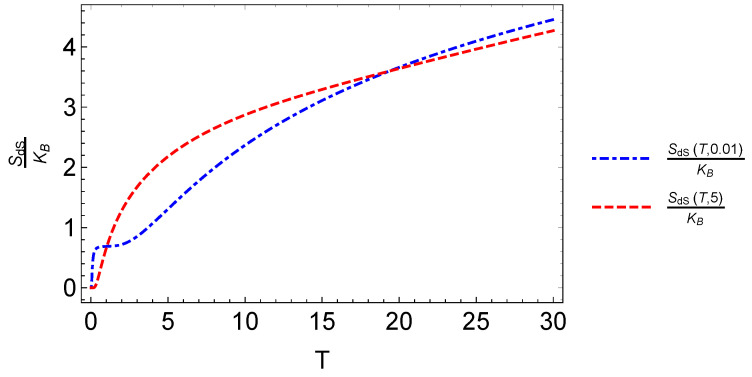
Entropy function $S_{dS}\left(T,B\right)$ of an electron with spin trapped in a quantum dot. We plot the entropy for two different values of the external magnetic field as a function of temperature. We use the characteristic value of $\omega_{d}=1.6407\times 10^{12}$ s^−1^. The dot-dashed line corresponds to the external magnetic field B = 0.01 T, and the dashed line corresponds to the value of B = 5 T.

We also see the interesting effect that the characteristic dot frequency plays in the transition between the direct MCE (ΔT>0) to the inverse behavior of the MCE (ΔT<0). For the lower values of $\omega_{d}$ (left and middle panel in Figure 10), a transition (change of sign) occurs at low temperatures. For the high value of $\omega_{d}$, we did not see a sign change and therefore always obtained the normal MCE (in the considered experimental range for the external magnetic fields). The aforementioned effect provides a form to control the type of MCE. If we focus on the results of the central panel of Figure 10, corresponding to $\hbar \omega_{d}∼1.07$ meV, we observe that the transition from the normal to the inverse case does not occur for all magnetic field values. Under 2.5 T, we do not observe an inverse MCE. Above 2.5 T, we get two zero points for the difference $-\Delta S_{dS}$ and therefore a small temperature region with inverse MCE. For example, when we calculated the entropy difference between $B_{f}=$ 5 T and $B_{i}=$ 0.01 T, the inverse MCE region obtained was approximately between 1.2 K and ∼18 K. This can be better understood if we plot the function $S_{dS}\left(T,B\right)$ for the two magnetic fields under discussion: $S_{dS}\left(T,0.01\right)$ and $S_{dS}\left(T,5\right)$, as can be appreciated in Figure 11.

In Figure 11, we appreciate the different regions that we obtain for −ΔS for the characteristic dot frequency of 1.07 meV. The entropy for a low magnetic field is not always greater than the corresponding one at a higher magnetic field. Between T=0.01 K and T=1 K, we have that $S_{dS}\left(T,0.01\right)>S_{dS}\left(T,20\right)$, then approximately from T=1.2 K to T=17.6 K, we have that $S_{dS}\left(T,0.01\right)<S_{dS}\left(T,20\right)$, and finally, from T=17.6 K onwards, we have the behavior $S_{dS}\left(T,0.01\right)>S_{dS}\left(T,20\right)$.

**Figure 12:**
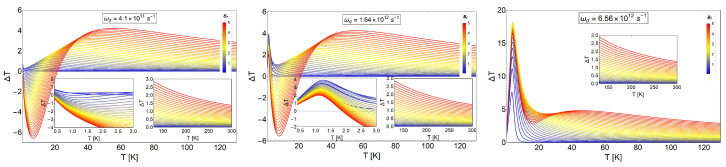
MCE effect for electrons with spin trapped in a quantum dot. ΔT as a function of temperature for different values of characteristic frequencies. The middle panel corresponds to a value of $\omega_{d}=1.6407\times 10^{12}$ s^−1^. The left panel corresponds to the value of $\frac{\omega_{d}}{4}$, and the right panel depicts the results using the value of $4\omega_{d}$. The range of the external magnetic field values, $B_{f}$, is between 0.02 T and 5 T, and $B_{i}=$ 0.01 T. The temperature range is from T=0.01 K to T=120 K. The insets show ΔT values zoomed in to a smaller range of temperatures and for near room temperature (left and middle panel).

In Figure 12, we display ΔT as a function of temperature. The three panel are in coherence with the values of −ΔS, as shown in Figure 11. We clearly see the behavior of ΔT from positive to negative and back to positive values as a function of temperature (left and middle panels). In particular, we highlight the middle panel, which shows a peak for ΔT with a value of approximately ΔT=4 K. This peak is higher as the value of the characteristic frequency of the dot increases, as we see for the extreme value for $\hbar \omega_{d}$ close to 4.8 meV in units of energy. Finally, another important discussion can be extracted from Figure 12: the effect of the parabolic trap in the oscillation that we observe for the MCE in the left and middle panel of that figure. We clearly observe a positive peak for ΔT close to zero temperature that can only be appreciated for $\hbar \omega_{d}\geq 1$ meV; under this value, it tends to disappear. The oscillation in MCE (sign change) was only obtained near the value of 1 meV for the quantum dot trap. For higher values of the trap frequency, we only observed the normal MCE for all the range of temperatures. Therefore, we can conclude that the strong energy trap of the quantum dots destroyed the oscillatory behavior of the MCE, and this conclusion is reinforced with the entropy differences in behavior shown in Figure 10.

We remark that the result presented for quantum dots with an intrinsic spin, when we obtain a different type of magnetocaloric response, can be used for adiabatic demagnetization refrigerators and magnetic field sensors [56]. It is important to note that our approach must be refined to take into account a many-electron scenario, which yields a more precise model. However, the non-interacting electron case is important due to its simplicity and the arising of a rich physics for comparatives cases. Moreover, these problems can be extended, for example, considering the edge states of the system. In particular, if we decrease the size of the dot radius (lower than the value of 20 nm), the edge states of the systems and their contributions will be significant in the results. These edge effects in the energy levels can be captured with the tight-binding approximation [57] (for example), and with these new levels of energy, we can construct the adequate thermodynamics and calculate the MCE for those cases. In addition, spin-orbit coupling can be taken into account in the formulation for a more realistic approach.

## 4. Conclusions

In this work, we explored the MCE for the non-degenerate and degenerate Landau problem and for an ensemble of non-interacting electrons (with and without intrinsic spin), where each one is trapped inside a semiconductor quantum dot modeled by a parabolic potential. We analyzed all the thermodynamics quantities and obtained the variation of the entropy and the temperature along the adiabatic strokes that characterize the MCE. In particular, we focused our investigation on the role of the degeneracy effects in MCE, finding that a strong degeneracy is related to an inverse MCE.

Finally, we showed that the inclusion of the spin term allowed us to find transitions of the MCE, from normal to the inverse case, due to the competition between the diamagnetism (case without spin) and the additional terms associated with paramagnetism (the case with spin). In terms of the degeneracy, we expected this result, due to the Zeeman split into the energy levels decreasing the degeneracy of the energy levels involved in the study, so the results must converge to normal MCE. Additionally, we can modify the characteristic frequency of the dots, to obtain a particular type of MCE (normal or inverse), allowing their use for a specific physical application (cooling or heating up).

References
